# TRPV1 regulates ApoE4-disrupted intracellular lipid homeostasis and decreases synaptic phagocytosis by microglia

**DOI:** 10.1038/s12276-023-00935-z

**Published:** 2023-02-01

**Authors:** Chenfei Wang, Jia Lu, Xudong Sha, Yu Qiu, Hongzhuan Chen, Zhihua Yu

**Affiliations:** 1 Department of Pharmacology and Chemical Biology, Shanghai Jiao Tong University School of Medicine, Shanghai, 200025 China; 2 Shanghai University of Traditional Chinese Medicine, Shanghai, 201203 China

**Keywords:** Dementia, Microglia

## Abstract

Although the ε4 allele of the apolipoprotein E (ApoE4) gene has been established as a genetic risk factor for many neurodegenerative diseases, including Alzheimer’s disease, the mechanism of action remains poorly understood. Transient receptor potential vanilloid 1 (TRPV1) was reported to regulate autophagy to protect against foam cell formation in atherosclerosis. Here, we show that ApoE4 leads to lipid metabolism dysregulation in microglia, resulting in enhanced MHC-II-dependent antigen presentation and T-cell activation. Lipid accumulation and inflammatory reactions were accelerated in microglia isolated from TRPV1^*flox/flox*^; Cx3cr1^cre^-ApoE4 mice. We showed that metabolic boosting by treatment with the TRPV1 agonist capsaicin rescued lipid metabolic impairments in ApoE4 neurons and defects in autophagy caused by disruption of the AKT-mTOR pathway. TRPV1 activation with capsaicin reversed ApoE4-induced microglial immune dysfunction and neuronal autophagy impairment. Capsaicin rescued memory impairment, tau pathology, and neuronal autophagy in ApoE4 mice. Activation of TRPV1 decreased microglial phagocytosis of synapses in ApoE4 mice. TRPV1 gene deficiency exacerbated recognition memory impairment and tau pathology in ApoE4 mice. Our study suggests that TRPV1 regulation of lipid metabolism could be a therapeutic approach to alleviate the consequences of the *ApoE4* allele.

## Alzheimer’s disease: Protein-activating chili extract improves brain’s immune function

A chili pepper extract that activates a sensory protein helps to improve lipid metabolism in immune cells in the brain, leading to better nerve cell health in a mouse model of Alzheimer’s disease. A team led by Zhihua Yu and Hongzhuan Chen from the Shanghai Jiao Tong University School of Medicine, China, showed that mice carrying the *ApoE4* risk factor gene for Alzheimer’s exhibited defects in how well their brain’s immune cells, known as microglia, processed lipids. These problems were exacerbated when the microglia lacked a working version of a sensory protein called TRPV1. Boosting the activity of this protein with capsaicin, a TRPV1-stimulating molecule found in hot chili peppers, reversed the metabolic defects, leading to improved microglial function, reduced Alzheimer’s-associated brain pathology and enhanced memory in the mice.

## Introduction

Among the ε2–ε4 polymorphic alleles, the ε4 allele of the apolipoprotein E (ApoE4) gene is the strongest genetic risk factor for late onset Alzheimer’s disease (LOAD)^1,2^, whereas inherited genetic mutations in amyloid precursor protein, presenilin 1, or presenilin 2 genes cause rarer early onset familial Alzheimer’s disease (AD)^3^. The *ApoE4* genotype lowers AD onset age and increases the risk of developing AD in a gene dose-dependent manner^2^. Beyond its role in regulating brain Aβ pathology and hyperphosphorylated tau, ApoE4 is also associated with multiple neurodegenerative disorders, including multiple sclerosis, Lewy body dementia, and Parkinson’s disease^4–6^.

The brain’s most prevalent lipid-binding protein, ApoE, transports cholesterol and phospholipids across organs in the periphery or between different cell types in the brain^7^, but its role in LOAD remains poorly understood. The dysregulation of lipid metabolism is commonly observed in ApoE4-affected neurological diseases^8^. ApoE4 disrupts endocannabinoid signaling and the neuroprotective function of sortilin in neuronal lipid metabolism^9^. ApoE4 also impairs insulin receptor trafficking by trapping it in endosomes in primary neurons. The interaction between ApoE4 and the insulin receptor leads to an impairment in neuronal insulin signaling and insulin-associated glycolysis and mitochondrial respiration^10^. ApoE4 impairs neuron-astrocyte coupling of fatty acid metabolism^11^. Lipid-poor ApoE4 was demonstrated to have a greater propensity to aggregate, which decreased the ATP binding cassette (ABC) transporter ABCA1 membrane recycling and ability to lipidate ApoE^12^.

Transient receptor potential vanilloid 1 (TRPV1), a ligand-gated nonselective cation channel, belongs to the transient receptor potential channel family^13^. TRPV1 is activated by capsaicin, the active component in hot chili peppers, as well as by several endogenous lipid molecules, such as anandamide. TRPV1 agonizts promote cholesterol efflux by upregulating the efflux of ABCA1 and ABCG1 in macrophages through liver X receptor α-dependent regulation of transcription in the pathogenesis of atherosclerosis. Capsaicin impeded foam cell formation by oxidized low-density lipoprotein in vascular smooth muscle cells (VSMCs) through autophagy induction^14^. Pharmacological activation of TRPV1 rescues Aβ-tolerant microglial metabolic impairment via the Akt/mechanistic pathway of rapamycin (mTOR) kinase pathway and restores immune function^15^. TRPV1 is expressed in sensory neurons, where it plays a key role in modulating synaptic transmission and plasticity^16^. TRPV1 activation alleviates the impairments in cognitive and synaptic plasticity via AMPAR endocytosis inhibition in an APP23/PS45 mouse model of AD^17^.

Here, we report that ApoE4, but not ApoE3, impairs neuronal and microglial lipid and energy metabolism in human ApoE-targeted replacement mice. ApoE4 leads to lipid metabolism dysregulation in microglia, resulting in enhanced MHC-II-dependent antigen presentation and T-cell activation. Lipid accumulation and inflammatory reactions were accelerated in microglia isolated from TRPV1^*flox/flox*^; Cx3cr1^cre^-ApoE4 mice. A high-fat diet (HFD) accelerated these effects in ApoE4 mice at middle age. These changes resulted in increased neuronal autophagy impairment and intracellular lipid droplet accumulation. We showed that TRPV1 activation with capsaicin reversed neuronal autophagy impairment and lipid metabolism dysfunction in primary neurons. Capsaicin rescued memory impairment, tau pathology, and neuronal autophagy in ApoE4 HFD mice. Activation of TRPV1 decreased neuronal lipid droplet accumulation and induced upregulation of microglial phagocytosis of synapses in ApoE4 HFD mice. TRPV1 gene deficiency exacerbated recognition memory and neuronal lipid homeostasis impairment in ApoE4 HFD mice. Our study suggests that TRPV1 regulation of neuronal lipid metabolism could be a therapeutic approach to alleviate the consequences of the *ApoE4* allele.

## Materials and methods

### Human brain samples

Human postmortem tissues from control and AD patients were obtained from the Netherlands Brain Bank (Netherlands Institute for Neuroscience, Amsterdam, Netherlands). Written informed consent for brain autopsy specimens for research purposes after death was obtained by the Netherlands Brain Bank. Immunofluorescence was performed using antibodies against Iba-1, NeuN, GFAP, and PLIN2 on 8-μm sections as described below.

### Animals

ApoE-targeted replacement mice in which the murine *ApoE* gene locus was replaced by the human *ApoE3* or *ApoE4* gene were a gift from Yu Qiu (Shanghai Jiao Tong University School of Medicine, Shanghai, China). TRPV1^−/−^ mice with a C57BL/6J background were a gift from Wei Huang (Department of Cardiology, The First Affiliated Hospital of Chongqing Medical University, Chongqing, China). ApoE mice were bred with TRPV1^−/−^ mice to generate TRPV1^+/−^ ApoE3, TRPV1^−/−^-ApoE3, TRPV1^+/−^-ApoE4, and TRPV1^−/−^-ApoE4 mice. All animals were housed under controlled conditions (12-h light-dark cycle and pathogen-free) and given free access to food and water. All animal procedures were approved by the Animal Experimentation Ethics Committee of Shanghai Jiao Tong University School of Medicine (Shanghai, China).

### Capsaicin treatment

Three-month-old male and female littermate ApoE3 and ApoE4 mice were fed a HFD (45% fat, Research Diets D12451, NJ, USA) or standard diet for 3 months. Capsaicin was purchased from Target Molecule (TargetMol, Corp., Shanghai, China). Five-month-old ApoE3 and ApoE4 HFD-fed mice were injected with capsaicin (1 mg/kg, intraperitoneally; one injection/day for one month)^18,19^. Animals were then subjected to behavioral tests.

### Novel object recognition test

A novel object recognition test was performed in a 40 × 40 × 35 cm acrylic cube open field box. On day one, mice were placed in the center of the empty open field box and allowed to habituate to the testing environment for 5 min. On day two, mice were placed into the open field box facing away from two identical objects and allowed to explore for 5 min. After a 4 h interval, mice explored the open field box for 5 min, but one object was replaced with a distinct novel object. Interactions with objects, including facing, sniffing, and/or touching, were recorded and analyzed in SuperMaze software. The percentage of time the mice spent exploring the novel object was calculated.

### Y maze spontaneous alternation

The Y maze spontaneous alternation test was performed in an apparatus consisting of three arms 40 cm long by 10 cm wide with walls 15 cm high. Each mouse was subjected to two trials. In the training trial, one arm was closed. Mice were placed into one arm (start arm) and explored in two arms for 5 min. After a 1 h interval, the closed arm was opened in the test trial as a novel arm. In the test trial, mice were placed into the start arm of the Y maze and allowed to explore for 5 min freely. The trials of mice were recorded and analyzed with SuperMaze software. The rate of spontaneous alternation (percent) was calculated by the ratio of the number of entries into the novel arm over total possible alterations minus 2 × 100.

### Morris water maze test

The Morris water maze was performed as previously described^20^. Briefly, tests were performed in a 120 cm diameter, 45 cm deep circular pool with a 4.5-cm-diameter escape platform 1 cm below the surface of the water. Opacified water was kept at 20 ± 1 °C. The procedures consisted of a 6-day hidden platform test and a 1-day probe trial. During the hidden platform test, the latency of mice to find and climb onto the platform was measured. After 6 days of training, the mice underwent a probe trial, in which the percent of time the mice spent in the target quadrant and the number of times the mice crossed the platform area were measured. Trials were recorded and analyzed by software from Shanghai Jialiang Software Technology Co, Ltd.

### Lipidomic analysis

For blood sample preparation, each aliquot (100 μL) of the plasma sample was thawed at 4 °C and mixed with 400 μL of cold methanol/acetonitrile (1:1, v/v) to remove protein. The mixture was centrifuged for 15 min (14,000*g*, 4 °C). The supernatant was dried in a vacuum centrifuge. For LC‒MS analysis, the samples were redissolved in 100 μL acetonitrile/water (1:1, v/v) solvent.

Untargeted LC‒MS/MS analyses were conducted using ultrahigh-performance liquid chromatography (1290 Infinity LC, Agilent Technologies, CA, USA) coupled to a quadrupole time-of-flight (AB Sciex TripleTOF 6600, CA, USA) at Nanjing Personalbio Technology Co., Ltd. Each sample was separated by both hydrophilic interaction chromatography and reversed-phase liquid chromatography with an electrospray ionization (ESI) source; a 2 μL aliquot of each sample was injected. For hydrophilic interaction chromatography separation, samples were analyzed using a 2.1 mm × 100 mm ACQUITY UPLC BEH 1.7 μm column (Waters, Wexford, Ireland). In both ESI-positive and -negative modes, the mobile phase contained A = 25 mM ammonium acetate and 25 mM ammonium hydroxide in water and B = acetonitrile. The gradient was 85% B for 1 min and was linearly reduced to 65% in 11 min, reduced to 40% in 0.1 min, maintained for 4 min, and then increased to 85% in 0.1 min, with a 5 min re-equilibration period. For reversed-phase liquid chromatography separation, a 2.1 mm × 100 mm ACQUITY UPLC HSS T3 1.8 μm column (Waters) was used. In ESI positive mode, the mobile phase contained A = water with 0.1% formic acid and B = acetonitrile with 0.1% formic acid; in ESI negative mode, the mobile phase contained A = 0.5 mM ammonium fluoride in water and B = acetonitrile. The gradient was 1% B for 1.5 min and was linearly increased to 99% in 11.5 min and maintained for 3.5 min. It was then reduced to 1% in 0.1 min with a 3.4 min re-equilibration period. Gradients were used at a flow rate of 0.3 mL/min, and column temperatures were maintained at 25 °C.

Untargeted data acquisition was performed in multiple reaction monitoring mode. The whole LC‒MS system was controlled by Agilent MassHunter Workstation software. The extracted multiple reaction monitoring data were integrated using Agilent MassHunter Quantitative Data Analysis.

### RNA-seq analysis

RNA isolation and purification from frozen mouse brains, cDNA library construction and sequencing were performed as previously described^21^. Briefly, total RNA was isolated from the hemibrains of mice with TRIzol reagent (Invitrogen 15596018, MA, USA) and treated with DNase to remove DNA. The quality and quantity of RNA were determined using a NanoDrop spectrophotometer (Thermo Scientific, MA, USA). mRNA was isolated from total RNA with poly-T oligonucleotide-attached magnetic beads. Ion interruption was used to interrupt RNA into fragments of approximately 300 bp. The first cDNA strand was synthesized by using 6-base random primers, after which the second-strand cDNA was synthesized by adding buffer solution, dNTPs, RNase H and DNA polymerase I. The RNA library was sequenced on the Illumina NovaSeq 5000 platform by Shanghai Personalbio Technology Co., Ltd. Differential expression of genes between groups was performed using the MAST algorithm of the Seurat package in R. Log_2_ (fold change) of the average expression of the genes in each group was generated. The adjusted *p* value was calculated using Bonferroni correction. Genes with an adjusted *p* < 0.05 and |fold change| > 1.5 were taken as differentially expressed genes. Hierarchical clustering of differentially expressed genes was performed using Heatmapper. Gene Ontology enrichment analysis and Kyoto Encyclopedia of Genes and Genomes enrichment analysis were performed to determine the main biological functions performed by the differentially expressed genes. Pathway analysis was performed using Metascape^22^.

### Western blotting

Western blotting was performed as previously described^15^. Mouse brain tissue or cultured cells were lysed on ice in radioimmunoprecipitation assay buffer (P0013B; Beyotime, Shanghai, China) with 1 mM phenylmethanesulfonylfluoride (ST506; Beyotime). Equal amounts of protein were loaded and separated by 8–15% sodium dodecyl sulfate‒polyacrylamide gel electrophoresis (Sangon, Shanghai, China) and transferred to polyvinylidene difluoride membranes (Millipore IPVH00010, ISEQ00010, Darmstadt, Germany). The membranes were blocked in 5% skim milk and immunoblotted with primary antibodies overnight at 4 °C. The following primary antibodies were used in this study: against p-IRS1 (Ser307) (Beyotime AI623 1:500, Shanghai, China), p-mTOR (Ser2448) (Cell Signaling Technology 5536 1:1000, MA, USA), p-Akt (Thr308) (Cell Signaling Technology 13038 1:1000, MA, USA), phospho-AMPKα (Thr172) (Cell Signaling Technology 2535 1:1000, MA, USA), β-actin (Beyotime AA125 1:1000, Shanghai, China), p-GSK3β (Ser9) (Cell Signaling Technology 5558 1:1000, MA, USA), and TRPV1 (Alomone ACC-030, Jerusalem, Israel). The membranes were then incubated with horseradish peroxide-conjugated anti-rabbit (7074; Cell Signaling Technology), anti-mouse (A0216; Beyotime), or goat anti-mouse 680 (A28183; Invitrogen, MA, USA) secondary antibodies. The blotted membranes were detected with an enhanced chemiluminescence detection system and scanned with an Odyssey CLx Imaging System (LI-COR).

### In vivo phagocytosis assay

FITC-labeled β-amyloid_1-42_ (FITC-Aβ_1-42_, GenicBio Limited, Shanghai, China) solution was mixed to a final concentration of 1 mg/mL. Mice were anesthetized and injected with 2 μl FITC-Aβ_1-42_ at a rate of 100 nL/min into the hippocampus (stereotaxic coordinates: −1.70-mm lateral, −0.70-mm anteroposterior, and −2.04-mm dorsoventral relative to the intersection of the coronal and sagittal suture). After 48 h, mice were perfused, and brain sections were harvested and prepared for immunostaining. A total of 13–16 visual fields in the hippocampus per animal were quantified to assess FITC-Aβ_1–42_ uptake by Iba-1^+^ microglia. The MCC of Iba-1 (red channel) to FITC-Aβ_1–42_ (green channel) was used to quantify the level of uptake.

### Immunofluorescence analysis

Paraffin-embedded human brain sections were deparaffinized with xylene, and antigen retrieval was performed with sodium citrate. Mouse brain sections were prepared as previously described^23,24^. Briefly, brain sections were blocked in phosphate-buffered saline (PBS) with 10% normal goat serum (NGS) and 0.3% Triton X-100 at room temperature (RT) for 30 min. The sections were incubated in PBS with 5% NGS, 0.3% Triton X-100, and primary antibodies at 4 °C overnight. The following primary antibodies were used in this study: Iba-1 (Wako 019-19741 1:200, Osaka, Japan), Iba-1 (Servicebio GB12105 1:500, Hubei, China), NeuN (Abcam 279296 1:1000, Cambridge, UK), NeuN (Abcam 279297 1:1000, Cambridge, UK), NeuN (Beyotime AF1072 1:100, Shanghai, China), GFAP (Beyotime AG259 1:50, Shanghai, China), PLIN2 (Abcam 108323 1:500, Cambridge, UK), Parkin (Beyotime AF7680 1:100, Shanghai, China), LC3B (Cell Signaling Technology 3868 1:100, MA, USA), MAP2 (Cell Signaling Technology 8707, 1:200, MA, USA), phospho-tau (Thr231) (Novus Biological 100-82249 1:200, CO, USA), phospho-tau (Ser202/Thr205) (Servicebio GB113883 1:500, Hubei, China), beta-2-microglobulin (Abcam 75853 1:250, Cambridge, UK), PSD95 (Cell Signaling Technology 3409 1:200, MA, USA), and CD68 (Abcam 53444 1:1000, Cambridge, UK). After primary antibody incubation, sections were washed in PBS and incubated in PBS with 5% NGS, 0.3% Triton X-100 and the following secondary antibodies: goat anti-mouse Alexa Fluor 488 (A32723), goat anti-rabbit Alexa Fluor 647 (A32733), goat anti-mouse Alexa Fluor 647 (A32728), goat anti-rabbit Alexa Fluor 555 (A32732, Invitrogen, MA, USA), and goat anti-rat Alexa Fluor 647 (Abcam 150159, Cambridge, UK) for 2 h at RT. For BODIPY staining, sections were incubated in PBS with BODIPY 493/503 (1 μg/mL, D3922, Invitrogen) for 15 min at RT. Immunofluorescent images were visualized and captured using a Leica TCS SP8 confocal laser scanning system (Leica Microsystems, Wetzlar, Germany). Quantification of the colocalization of antibodies against PLIN2/NeuN, PLIN2/Iba-1, BODIPY/Parkin, and BODIPY/LC3 was performed using ImageJ software (NIH, MD, USA), as described previously^15^, at 120-μm intervals of six sections per brain in a blinded manner. The average value of each mouse brain was calculated.

Measurement of granule cell layer thickness was described previously^25^. Briefly, three sections (around bregma −1.7 mm and determined using mouse brain stereotaxic coordinates^26^) from each mouse were stained with NeuN/DAPI. The thickness of the dentate gyrus granular cell layer was measured by drawing a scale perpendicular to the cell layer at two spots in all three sections and taking the average of each mouse.

### Three-dimensional (3D) reconstruction of confocal images

Confocal image stacks of BODIPY^+^ microglia or PSD95-engulfed microglia with surface-rendering and spot-rendering were obtained with Imaris Bitplane 9.6.1 software as previously described^1^. Briefly, to quantify the number of PSD95 puncta in each group of mice, detected PSD95 puncta were automatically counted using Imaris 9.6.1 software. For quantification of the number of PSD95 puncta engulfed in Iba-1^+^ microglia, the PSD95 puncta previously detected were automatically counted within the Iba-1^+^ surface software. Five random fields per animal were quantified.

### Microglial morphological analysis

Two-dimensional morphological analysis of microglia was performed using ImageJ as described previously^20^. Briefly, 40 microglial cells per group randomly selected from the cerebral cortex were segmented into single cells using a custom-written ImageJ plugin. Single-cell images were automatically converted to 8-bit and transformed into binary images by application of an automatically calculated intensity threshold. The parameters “Number of branches”, “Number of junctions”, and “Mean branch length” were quantified using the FIJI plugins “Skeletonize” and “Analyze skeleton 2D/3D” to skeletonize and analyze the binary single-cell images obtained in the previous step^27^.

### Cell culture

Cerebral cortices were harvested from newborn (P0–P2) C57BL/6 mice as previously described^15^. Neurons were cultured with 150 nM human recombinant ApoE3 (PeproTech 350-02, NJ, USA) or ApoE4 (PeproTech 350-04) combined with 100 μM PA for 24 h. In some experiments, neurons were preincubated with 10 μM capsaicin for 30 min followed by the addition of ApoE3 or ApoE4 and PA. Human neuroblastoma-derived SH-sy5y cells were exposed to 150 nM human recombinant ApoE3 or ApoE4 combined with 100 μM PA for 24 h. Microglia-conditioned medium (MCM) was collected from the culture medium of human recombinant ApoE3- and ApoE4-treated BV2 cells. BV2 cells were preincubated with 10 μM capsaicin for 30 min, and then ApoE3 or ApoE4 was added. DIV8 neurons were incubated with 95% neuronal medium and 5% MCM at 37 °C with 5% CO_2_ for 24 h.

### Mitochondrial membrane potential

Mitochondrial membrane potential (MMP) was measured by JC-1 (Beyotime C2005). Cells were incubated with 10 μM JC-1 for 20 min at 37 °C. The fluorescence level was measured with a Thermos Multiskan^TM^ microplate reader. Red and green fluorescence intensities were measured separately at Ex/Em = 560/590 nm and 480/530 nm, respectively.

### Measurement of reactive oxygen species

Cells were loaded with 10 μM 5-(and-6)-chloromethyl-2′,7′-dichlorodihydrofluorescein diacetate (CM–H_2_DCFDA, S0033S, Beyotime) to measure ROS generation as previously described^15^. Briefly, cells were incubated with DCFH-DA for 20 min at 37 °C, and ROS were measured with a fluorescence microplate reader (Thermo Fisher) with DCFH-DA at a wavelength of 450 nm.

### Measurement of mitochondrial respiration

Mitochondrial respiration was measured by directly detecting the oxygen consumption rate with a Seahorse XFe cell analyzer (Agilent Technologies) as previously described^20^. Briefly, cells were plated in XF96 plates at 5 × 10^5^ cells/well. After adherence overnight and treatment with human recombinant ApoE3 or ApoE4, cells were washed and incubated in Seahorse XF Base medium (Seahorse Bioscience 102353-100, CA, USA) supplemented with 25 mM glucose, 200 mM glutamine, and 1 mM pyruvate. The oxygen consumption rate was determined using 5 μM oligomycin, 10 μM carbonyl cyanide p-(trifluoromethoxy) phenylhydrazone, 10 μM rotenone, and antimycin A to measure mitochondrial respiration. The results were analyzed and exported using Wave version 2.6.0 (Agilent Technologies).

### Immunocytochemistry

Primary neurons were treated with a solution of MitoTracker Red CMXRos (Beyotime C1035 200 nM, Shanghai, China) for 17 min at 37 °C. Cells were then fixed with 4% PFA for 10 min at RT and blocked in PBS containing 10% NGS and 0.3% Triton X-100 for 30 min at RT. Cells were incubated in the following primary antibodies overnight at 4 °C: anti-LC3B (83506, Cell Signaling Technology), anti-Parkin (AF7680; Beyotime), and anti-MAP2 (8707, Cell Signaling Technology). Cells were incubated in the following secondary antibodies for 1 h at RT: goat anti-mouse Alexa Fluor 647 (A32728, Invitrogen) and goat anti-rabbit Alexa Fluor 647 (A32733, Invitrogen). Images were taken with a Leica TCS SP8 confocal laser scanning system.

### Phagocytosis assay

BV2 cells were plated at a density of 5 × 10^4^ cells/well on 1.5-mm^2^ coverslips in DMEM with 10% FBS and treated with human recombinant ApoE3 or ApoE4 for 24 h. Amine-modified polystyrene latex beads (Sigma L2778, Darmstadt, Germany) were added at a concentration of 5 μl/ml per well for 4 h at 37 °C. The cells were washed with PBS to remove the nonphagocytized beads and fixed with 4% paraformaldehyde for 10 min. Phagocytosis of the beads by BV2 cells was observed under a Leica TCS SP8 confocal laser scanning system (Leica Microsystems, Wetzlar, Germany). Fifteen randomly selected visual fields per group (maximum projection of the z-stack across the whole section) were photographed by a Leica TCS SP8 confocal scanning laser microscope with LasX software 3.2.

### Flow cytometry

Mononuclear cells were isolated from the brain tissues of 15-month-old ApoE3 and ApoE4 mice. Briefly, brain tissues were digested with 1 mg/mL collagenase IV (Sigma C4-28, Darmstadt, Germany) and 20 μg/mL DNAse I (Sigma D5025, Darmstadt, Germany) diluted in DMEM (Bioagrio, Shanghai, China). Mononuclear cells were separated with a 70 μm cell strainer. For cell staining, isolated cells were stained with antibodies against CD45 (clone 30-F11, BioLegend 103154, California, USA), CD11b (clone M1/70, BioLegend 101211), MHC-II (clone M5/114.15.2, BioLegend 107608), TCRβ (clone H57-597, BioLegend 109205), CD4 (clone RM4-4, BioLegend 116022), and CD8a (clone 53-6.7, BioLegend 100722), and 7-AAD cellular viability dye (Yeasen 40745ES64, Shanghai, China) for 30 min at room temperature. Samples were analyzed by flow cytometry using an Attune Nxt flow cytometer (Thermo Fisher) and FlowJo software (Tree Star, Ashland, OR, USA).

### Data analysis

Data collection was randomized for all experiments. Experimenters were blinded for imaging and analyses. Statistical analyses were performed using GraphPad Prism 8.0. Statistical significance was determined by two-tailed Student’s *t test* for paired comparisons and one-way ANOVA with post hoc Tukey’s test for multiple comparisons. Data are presented as the means ± standard errors of the means. Statistical parameters are detailed in the legend for each figure. *P* values less than 0.05 were considered statistically significant.

## Results

### The ApoE4 allele led to lipid metabolism dysfunction in neurons and microglia

**Fig. 1:**
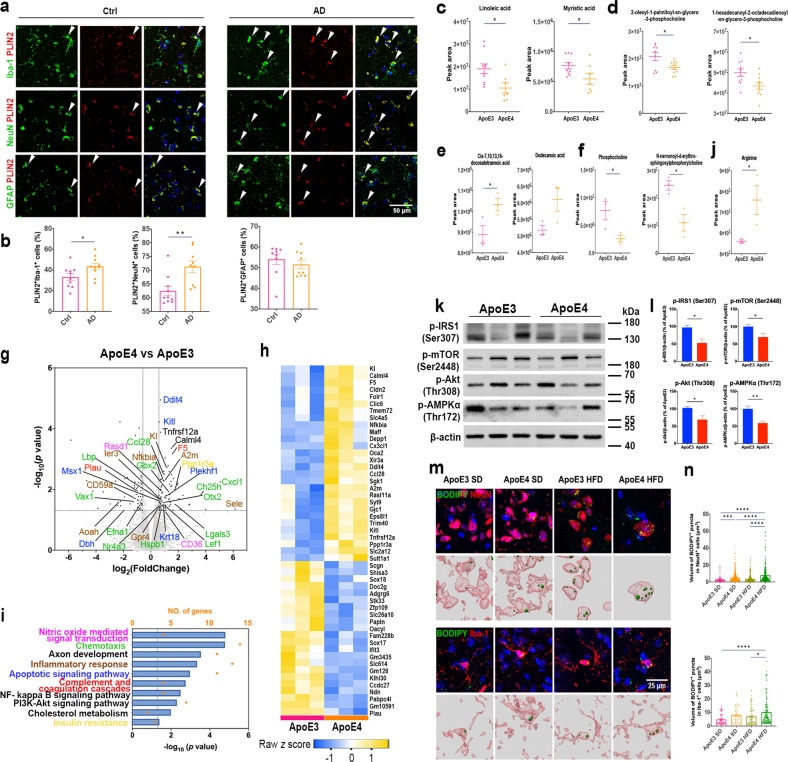
APOE4 allele led to lipid metabolism dysfunction in neurons and microglia. **a** Representative immunofluorescent images of PLIN2^+^ lipid droplets, Iba-1^+^ microglia, NeuN^+^ neurons, and GFAP^+^ astrocytes in the cerebral cortex of normal and ApoE4 AD individuals. **b** Mander’s colocalization coefficient quantification of PLIN2 and Iba-1, NeuN, or GFAP. **c**, **d** Significantly changed metabolites in the peripheral plasma of ApoE3 and ApoE4 mice. **e**, **f** Significantly changed metabolites in ApoE3 and ApoE4 mouse brains. **g** Volcano plot showing differentially expressed genes in ApoE3 and ApoE4 mouse brains. The dotted lines indicate the *p* < 0.05 and |fold change| > 1.5 cutoffs (two-sided Student’s *t* test, Benjamini‒Hochberg FDR). Genes enriched in pathways are colored in the same color in (**i**). **h** Heatmap showing the top 50 differentially expressed genes between ApoE3 and ApoE4 mice. **i** Enrichment pathway analysis of genes differentially expressed between ApoE3 and ApoE4 mouse brains. **j** Arginine levels in ApoE3 and ApoE4 mice were analyzed by MS. **k**, l Immunoblotting and quantification showing p-IRS1 (Ser307), p-mTOR (Ser2448), p-Akt (Thr308), and p-AMPK in ApoE3 and ApoE4 mouse brains. **m** Immunofluorescent images in the upper panel show brain cortical sections of ApoE3 and ApoE4 mice fed a SD or HFD costained for BODIPY^+^NeuN^+^ or BODIPY^+^Iba-1^+^ cells. The lower panel shows 3D reconstructions of BODIPY^+^NeuN^+^ or BODIPY^+^Iba-1^+^ cells. **n** Quantification of BODIPY^+^NeuN^+^ and BODIPY^+^Iba-1^+^ cell numbers in the cerebral cortex. *n* = 3 mice per group. Experiments on primary neurons were performed three times in technical triplicates. Statistical tests: two-sided Student’s *t* test (**b**–**f**, **j**, **l**). One-way ANOVA followed by Tukey’s post hoc test (**n**). Data represent the mean ± s.e.m. *p < 0.05, **p < 0.01, ***p < 0.001, ****p < 0.0001. Scale bars, 50 μm (**a**); 100 μm (**p**).

Genome-wide association studies have indicated that lipid metabolic disorders are involved in a number of late-onset neurodegenerative diseases, such as AD^28–30^. Lipid droplets are cytoplasmic organelles that store triacylglycerides as well as other neutral lipids, such as cholesterol esters, regenerating fatty acids (FAs) for energetic needs^31,32^. Here, postmortem brain cerebral cortex tissue sections from AD patients carrying the *ApoE4* allele and those from age-matched normal individuals were first costained with the lipid droplet surface protein perilipin 2 (PLIN2) and the microglial marker Iba-1, the neuronal marker NeuN, or the astrocyte marker GFAP. Confocal images showed more colocalization of PLIN2^+^ lipid droplets and Iba-1^+^ microglia as well as NeuN^+^ neurons, suggesting that AD patients carrying the *ApoE4* allele accumulated more lipid droplets in microglia and neurons than normal individuals (Fig. 1a, b). However, lipid droplet accumulation in astrocytes did not differ between AD patients carrying the *ApoE4* allele and normal individuals (Fig. 1a, b).

Mass spectrometry (MS) was used to quantify metabolites in the peripheral serum of ApoE4 and ApoE3 mice. Our results revealed significant downregulation of several long-chain and medium-chain FAs (linoleic acid and myristic acid) (Fig. 1c) as well as some phosphocholines (PCs) (Fig. 1d) in the peripheral serum of ApoE4 mice compared with ApoE3 mice. In the brain tissues of ApoE4 and ApoE3 mice, MS revealed significant upregulation of cis-7,10,13,16-docosatetraenoic acid (adrenic acid, an ω-6 polyunsaturated FA) and dodecanoic acid (lauric acid, a medium chain FA) in ApoE4 mice compared with ApoE3 mice (Fig. 1e). Wang et al. identified downregulated linoleic acid and myristic acid as new AD biomarkers, as AD patients exhibited lower levels of identified FAs in the peripheral serum and higher levels of identified FAs in the brain than age-matched controls^33,34^. The significant upregulation of the transfer of FAs from the blood to the brain may be due to blood‒brain barrier damage in AD patients^35^. Increasing adrenic acid and dodecanoic acid were reported to have proinflammatory effects and induce oxidative stress^36,37^. MS also showed significant downregulation of PC (phosphocholine and N-nervonoyl-d-erythro-sphingosylphosphorylcholine) in ApoE4 mouse brains (Fig. 1f). Varma et al. demonstrated that AD patients showed lower levels of PC in both plasma and brain samples than normal individuals^38^. Downregulation of PC in ApoE4 mouse brains suggested disruption of intracellular lipid homeostasis^39^.

Unsupervised cluster analysis segregated ApoE3 from ApoE4 mice and revealed prominent differences between their transcriptomes; 76 significantly differentially expressed genes were detected (Fig. 1g, h). Enrichment analysis showed pathways involved in the inflammatory response (*CXCL1*, *GBX2*, *HSPB1*, *LBP*, *NFKBIA*, and *A2M*). In addition, several energy metabolic processes, such as the PI3K-Akt pathway (*EPHA2*, *FLT4*, *ITGA5*, and *LPAR6*), cholesterol metabolism (*APOE* and *CD36*), and insulin resistance (*FOLR1*, *CALM14*, and *PPP1R3A*), were enriched in ApoE4 mice (Fig. 1i). These enriched pathways indicated dysregulated energetic metabolism in ApoE4 mouse brains. Previous studies showed that fatty acid translocase (CD36), a key FA transport protein, was upregulated in ApoE4 mouse brains compared with ApoE3 mice, indicating that upregulation of FA transport likely explained the higher FA levels in ApoE4 mouse brains^39^. Moreover, MS results indicated significant upregulation of arginine expression in ApoE4 mouse brains compared with ApoE3 mouse brains (Fig. 1j). Increased arginine levels indicate more nitric oxide production, consistent with “nitric oxide-mediated signal transduction” pathway enrichment in the ApoE4 mouse brain (Fig. 1i). Immunoblotting showed lower protein levels of Akt/mTOR and phosphorylated insulin receptor substrate 1 (p-IRS1) in ApoE4 mice than in ApoE3 mice (Fig. 1k, l), suggesting that ApoE4 led to energy metabolism dysfunction and insulin resistance.

To investigate the impact of the *ApoE4* allele on the lipid metabolism of the mouse brain, 3-month-old humanized ApoE3- or ApoE4-knockin mice were subjected to a standard diet or HFD for 3 months. Immunofluorescence costaining of NeuN^+^ neurons, Iba-1^+^ microglia, or GFAP^+^ astrocytes with BODIPY was applied to determine the localization of lipid droplets. Micrographs and three-dimensional (3D) reconstruction images revealed that lipid droplets significantly accumulated in both neurons and microglia in ApoE4 HFD mice compared with ApoE3 HFD mice (Fig. 1m, n).

### TRPV1 activation reversed ApoE4-induced microglial lipid droplet accumulation and immune dysfunction

**Fig. 2:**
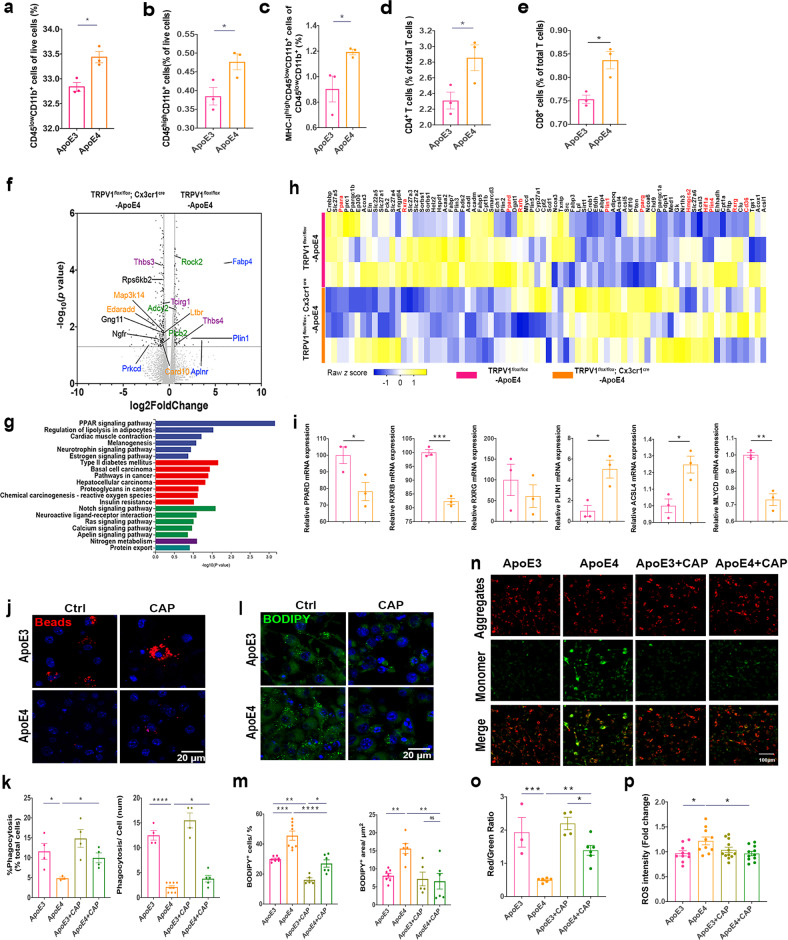
TRPV1 activation reversed ApoE4-induced microglial lipid droplet accumulation and immune dysfunction. **a** Gating strategy for flow cytometric analysis of ApoE3 and ApoE4 mouse brains. **b**–**f** Quantification of flow cytometry in ApoE3 and ApoE4 mouse brains displaying the proportion of resident microglia (CD45^low^CD11b^+^), monocytes (CD45^high^CD11b^+^), CD4^+^ T cells, CD8^+^ T cells, and MHC-II high-expressing resident microglia. **g** Volcano plot showing differentially expressed genes between TRPV1^*flox/flox*^; Cx3cr1^cre^-ApoE4 and TRPV1^*flox/flox*^-ApoE4 mouse brains. The dotted lines indicate the *p* < 0.05 and |fold change| > 1.5 cutoff (two-sided Student’s *t* test, Benjamini‒Hochberg FDR). Genes enriched in pathways related to lipid accumulation are colored blue, those enriched in pathways related to the immune response are colored purple, those enriched in pathways related to inflammatory reactions are colored orange, and those enriched in pathways related to chemotaxis are colored green. **h** KEGG enrichment pathway analysis of genes differentially expressed between TRPV1^*flox/flox*^; Cx3cr1^cre^-ApoE4 and TRPV1^*flox/flox*^-ApoE4 mouse brains. Blue indicates the organismal systems category, red indicates the human diseases category, green indicates the environmental information processing category, purple indicates the metabolism category, and cyan indicates the genetic information processing category. **i** Heatmap showing the expression levels of PPAR target genes between TRPV1^*flox/flox*^; Cx3cr1^cre^-ApoE4 and TRPV1^*flox/flox*^-ApoE4 mouse brains. **j** Histograms depict quantitative analysis of differentially expressed target genes of PPARs between TRPV1^*flox/flox*^; Cx3cr1^cre^-ApoE4, and TRPV1^*flox/flox*^-ApoE4 mouse brains. **k**–**p** BV2 cells treated with human recombinant ApoE3 or ApoE4 (150 nM) with or without 10 μM capsaicin pretreatment. **k**, **l** Representative confocal images and quantification of the phagocytosis assay. **m**, **n** Representative confocal images and quantification of BODIPY staining in BV2 cells. **o**, **p** Quantification of cellular MMP (**o**) and cellular ROS (**p**) production in BV2 cells stained by JC-1 and DCFH-DA. *n* = 3 mice per group. Experiments on BV2 cells were performed three times in technical triplicates. Statistical tests: two-sided Student’s *t* test (**b**–**f**, **j**) and one-way ANOVA followed by Tukey’s post hoc test (**h**, **i**, **l**, **o**, **n**, **p**). Data represent the mean ± s.e.m. *p < 0.05, **p < 0.01, ***p < 0.001, ****p < 0.0001. Scale bars, 20 μm (**k**, **m**), 100 μm (**o**).

To investigate the effects of ApoE4 on immune cells in the mouse brain, flow cytometry was used to quantify immune cells in ApoE3 and ApoE4 mouse brains (Fig. 2a–e, Supplementary Fig. 1). Flow cytometry showed that CD45^low^CD11b^+^ microglia were significantly increased in ApoE4 mouse brains compared to those in ApoE3 mouse brains (Fig. 2a). MHC-II^high^CD45^low^CD11b^+^ microglia were significantly increased in ApoE4 mouse brains compared to those in ApoE3 mouse brains (Fig. 2c). Peripheral immune cells, CD45^high^CD11b^+^ monocytes, CD4^+^ T cells, and CD8^+^ T cells were upregulated in ApoE4 mice compared to those in ApoE3 mice (Fig. 2b, d, e). Upregulation of MHC-II^high^ microglia expression indicated neuroinflammation-induced peripheral adaptive immune cell infiltration into the brain^40^.

We investigated whether TRPV1 contributed to immune dysregulation of microglia in the ApoE4 mouse brain. TRPV1^*flox/flox*^; Cx3cr1^cre^-ApoE4 and TRPV1^*flox/flox*^-ApoE4 mouse brain tissues were analyzed by RNA-seq. Unsupervised cluster analysis segregated TRPV1^*flox/flox*^-ApoE4 from TRPV1^*flox/flox*^; Cx3cr1^cre^-ApoE4 mice and revealed 420 significantly differentially expressed genes (Fig. 2f). Enrichment analysis showed pathways involved in lipid accumulation (*FABP4*, *PRCKD*, *PLIN1*, and *APLNR*), immune response (*THBS3*, *TETRG1*, and *THBS4*), inflammatory reactions (*MAP3KL4*, *EDARADD*, *LBR*, and *CARD10*), and chemotaxis (*ROCK2*, *ADOY2*, and *PLCB2*) (Fig. 2g). KEGG enrichment analysis revealed that the peroxisome proliferator-activated receptor (PPAR) signaling pathway was the most significantly enriched pathway in TRPV1^*flox/flox*^; Cx3cr1^cre^-ApoE4 mice compared to TRPV1^*flox/flox*^-ApoE4 mice (Fig. 2h). Then, the transcript levels of 78 PPAR target genes were observed, and the results showed that *PPARD* and retinoic X receptor (*RXRG*) were significantly downregulated in TRPV1^*flox/flox*^; Cx3cr1^cre^-ApoE4 mice compared to those in TRPV1^*flox/flox*^-ApoE4 mice (Fig. 2i). *PPARG* in TRPV1^*flox/flox*^; Cx3cr1^cre^-ApoE4 mice also showed a trend toward downregulation (Fig. 2i). Analysis of PPAR target genes also revealed that genes related to lipid accumulation (*PLIN1* and *ACSL4*) were upregulated in TRPV1^*flox/flox*^; Cx3cr1^cre^-ApoE4 mice compared to those in TRPV1^*flox/flox*^-ApoE4 mice, while a gene related to lipid efflux (*MLYCD*) was downregulated in TRPV1^*flox/flox*^; Cx3cr1^cre^-ApoE4 mice (Fig. 2i). Our transcriptome analysis results indicated that lipid accumulation in microglia is downstream of TRPV1 activation in the ApoE4 mouse brain.

Amine-modified polystyrene latex beads were used to validate the role of TRPV1 in ApoE4 microglial phagocytosis. Less fluorescent latex bead phagocytosis was observed in ApoE4 BV2 cells than in ApoE3 cells, while capsaicin-treated ApoE4 cells phagocytosed more fluorescent beads than ApoE4 BV2 cells (Fig. 2j, k). Immunofluorescence showed that capsaicin decreased the BODIPY^+^ puncta in ApoE4 BV2 cells in a dose-dependent manner (Fig. 2l, m, Supplementary Fig. 2). JC-1 and DCFH-DA were used to analyze MMP or cellular ROS production in BV2 cells. Pharmacological activation of TRPV1 with capsaicin attenuated MMP depolarization (Fig. 2n, o) and ROS production in ApoE4 BV2 cells (Fig. 2p).

### TRPV1 activation reversed ApoE4-induced neuronal metabolism impairment and lipid droplet accumulation

**Fig. 3:**
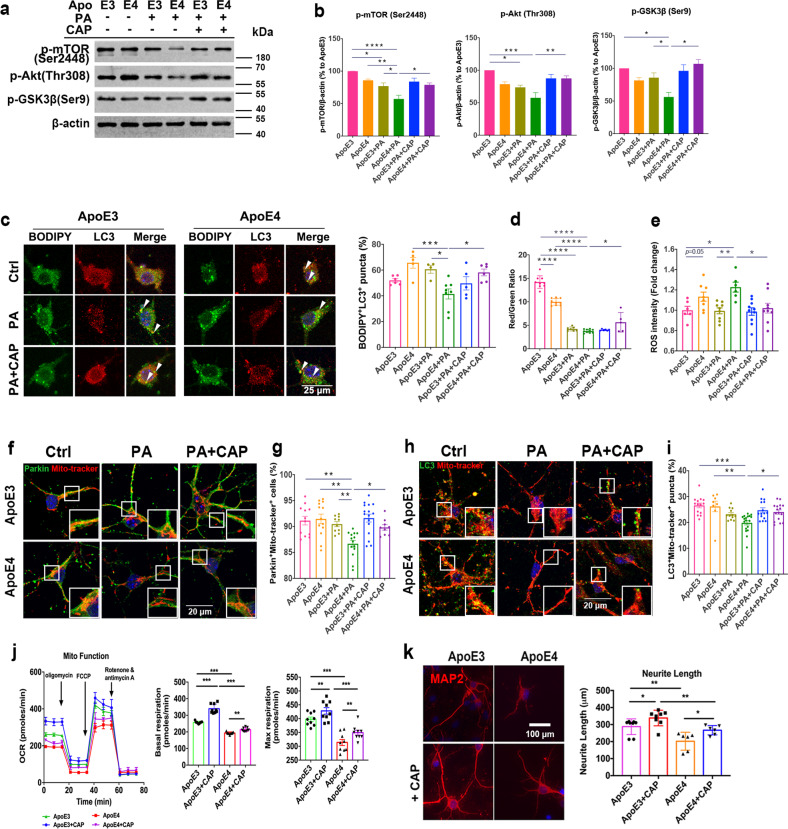
TRPV1 activation reversed ApoE4-induced neuronal autophagy impairment and lipid droplet accumulation in vitro. **a**, **b** Western blot and quantification of phospho-mTOR (Ser2448), phospho-Akt (Thr308), and phospho-GSK3β in SH-sy5y cells treated with 150 nM recombinant ApoE and 100 μM PA for 24 h. **c** Representative immunofluorescent images and quantification of BODIPY^+^LC3^+^ puncta in primary neurons. **d** Quantification of cellular MMP in primary neurons stained with JC-1. **e** Quantification of cellular ROS production in primary neurons stained with DCFH-DA. **f**–**i** Representative immunofluorescent images and quantification of Parkin and MitoTracker Red (**f**, **g**) or LC3 and MitoTracker Red (**h**, **i**). **j** Mitochondrial stress test of 150 nM ApoE3- and ApoE4-treated neurons for 24 h with pretreatment with 10 μM capsaicin; basal respiration and maximal respiration are shown. **k** Confocal images of MAP2^+^ (neurite) cells treated with 150 nM ApoE3 and ApoE4 for 24 h. Quantification of the mean neurite length of MAP2^+^ cells from confocal images. Experiments on primary neurons were performed three times in technical triplicates. Statistical tests: one-way ANOVA followed by Tukey’s post hoc test (**b**–**e**, **g**, **i**, **j**, **k**). Data represent the mean ± s.e.m. *p < 0.05, **p < 0.01, ***p < 0.001, ****p < 0.0001. Scale bars, 25 μm (**c**), 20 μm (**f**, **h**), 100 μm (**k**).

SH-sy5y cells and primary cortical neurons were exposed to 150 nM human recombinant ApoE3 or ApoE4 combined with 100 μM palmitic acid (PA) for 24 h. PA is a saturated long-chain fatty acid that can be used to mimic HFD exposure in vivo^41^. Immunoblotting showed that the Akt/mTOR signaling pathway was significantly decreased following PA-treated ApoE4 SH-sy5y cells, and was rescued by 10 μM capsaicin pretreatment (Fig. 3a, b). Immunofluorescence showed that capsaicin increased the number of BODIPY^+^LC3^+^ puncta in PA-treated ApoE4 neurons (Fig. 3c). Increased colocalization of BODIPY and LC3B puncta indicated that the TRPV1 agonist promoted neuronal lipid droplet clearance by inducing autophagy.

JC-1 and DCFH-DA were used to analyze MMP or cellular ROS production in primary neurons. Pharmacological activation of TRPV1 with capsaicin attenuated MMP depolarization and ROS production in PA-treated ApoE4 neurons (Fig. 3d, e). Primary cortical neurons were costained with the mitochondrial marker MitoTracker and the autophagy marker Parkin or LC3B. The colocalization of mitochondria with Parkin or LC3B puncta was significantly decreased in PA-treated ApoE4 neurons (Fig. 3g, i). Capsaicin treatment increased the colocalization of mitochondria with Parkin or LC3B puncta in PA-treated ApoE4 neurons, indicating that TRPV1 activation rescued ApoE4-induced neuronal autophagy impairment during lipid metabolism (Fig. 3f-i).

Capsaicin treatment significantly increased basal and maximal respiration in ApoE4 + capsaicin primary neurons compared to that in ApoE4 cells (Fig. 3j). Immunofluorescent images of MAP2^+^ neurons showed that the length, branch counts, and area of neurites were significantly longer in ApoE4 + capsaicin neurons than in ApoE4 neurons (Fig. 3k).

### TRPV1 activation rescued memory impairment and neuron loss in ApoE4 HFD-fed mice

**Fig. 4:**
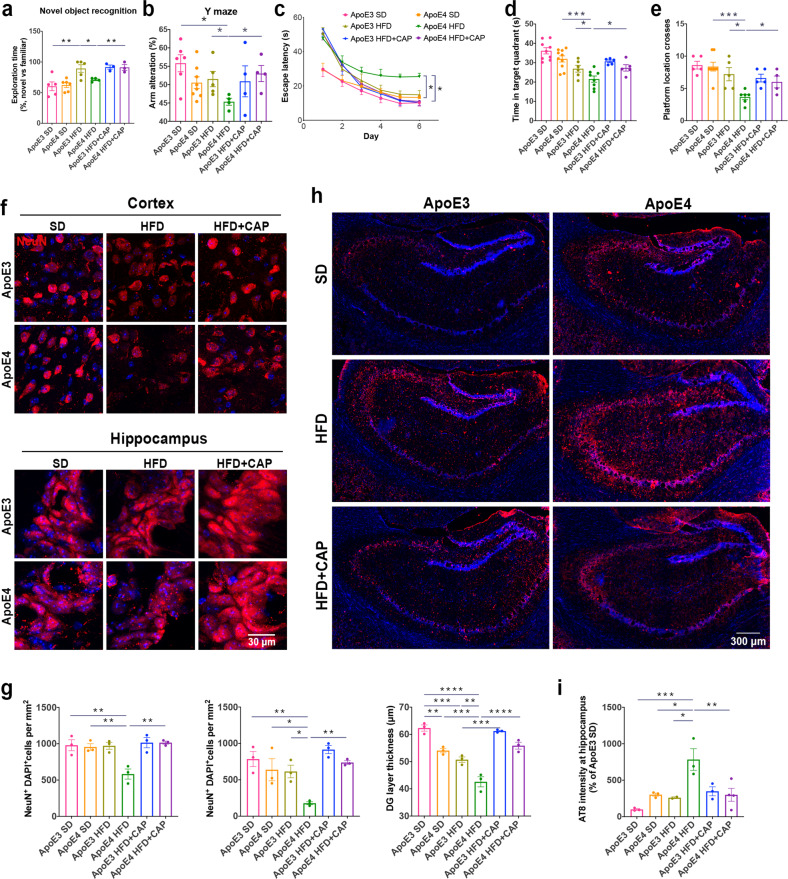
TRPV1 activation rescued memory impairment and neuronal loss in ApoE4 HFD-fed mice. **a**, **b** Novel object recognition and Y maze behavioral assessments for ApoE3 and ApoE4 HFD-fed mice with capsaicin treatment. **c**–**e** MWM behavioral assessment of ApoE3 and ApoE4 mice. **c** Line chart shows MWM escape latency (time to find the hidden platform) for six consecutive days. Histograms show the time spent in the target quadrant (**d**) and the number of platform location crossings (**e**) in the MWM probe trial. *n* = 5, 7, 5, 5, 4, 4 mice per group during behavioral assessments (**a**–**e**). **f**, **g** Representative immunofluorescent images and quantification of NeuN^+^ cells in the cerebral cortex and hippocampus of ApoE3 and ApoE4 HFD-fed mice treated with capsaicin. **h**, **i** Phosphorylated tau (AT-8)-covered area in the hippocampus of ApoE3, ApoE3 HFD-fed, ApoE4, and ApoE4 HFD-fed mice treated with capsaicin. *n* = 3 mice per group (**f**, **h**). Statistical tests: one-way ANOVA followed by Tukey’s post hoc test (**a**, **b**, **d**, **e**, **g**, **i**) and two-way ANOVA followed by Tukey’s post hoc test (**c**). Data represent the mean ± s.e.m. *p < 0.05, **p < 0.01, ***p < 0.001, ****p < 0.0001. Scale bars, 30 μm (**f**); 300 μm (**h**).

We then monitored whether TRPV1 activation could rescue neuronal lipid metabolism in vivo. Briefly, 3-month-old male and female ApoE3 and ApoE4 mice were fed a HFD for 3 months. Five-month-old ApoE3 and ApoE4 HFD mice were treated with 1 mg/kg capsaicin by intraperitoneal injection for 1 month, followed by behavioral assessments, including novel object recognition, Y maze, and Morris water maze (MWM) testing. ApoE4 HFD mice showed less preference for the novel object than ApoE3 HFD mice; this effect was rescued by capsaicin treatment (Fig. 4a, Supplementary Fig. 3a). Spatial working memory functions of mice were analyzed by the Y maze test^42^. The spontaneous alteration analysis revealed impaired memory of ApoE4 HFD mice compared with ApoE3 HFD mice, while capsaicin-treated ApoE4 HFD mice spent more time exploring the novel arm than did ApoE4 HFD mice (Fig. 4b). In the MWM, the learning ability of ApoE4 HFD mice was significantly impaired compared with that of ApoE3 HFD mice. Capsaicin treatment significantly improved the learning ability of ApoE4 HFD mice (Fig. 4c). Probe trial results were conducted after the last training session. Capsaicin treatment significantly increased the time in the target quadrant and platform location crosses of ApoE4 HFD + capsaicin group mice compared with ApoE4 HFD mice (Fig. 4d, e). These behavioral assessments indicated improved learning and memory capacity in capsaicin-treated ApoE4 HFD mice.

The density of NeuN^+^ neurons in the cortex and CA1 in the hippocampus was significantly lower in ApoE4 HFD mice than in ApoE3 HFD mice, while the density of NeuN^+^ cells was rescued in ApoE4 HFD + capsaicin mice compared with ApoE4 HFD mice (Fig. 4f, g). The granule cell layer in the dentate gyrus was also significantly thicker in ApoE4 HFD + capsaicin mice than in ApoE4 HFD mice (Fig. 4g, Supplementary Fig. 3b). We compared the relative phosphorylated tau pathology by immunostaining of phosphorylated tau (AT8). Immunofluorescent images of phosphorylated tau (AT8) revealed significant upregulation in the hippocampus of ApoE4 HFD mice compared with ApoE3 HFD mice. Pharmacological activation of TRPV1 with capsaicin ameliorated ApoE4 HFD-induced tau pathology, as determined by immunostaining of phosphorylated tau in the hippocampus (Fig. 4h, i).

**Fig. 5:**
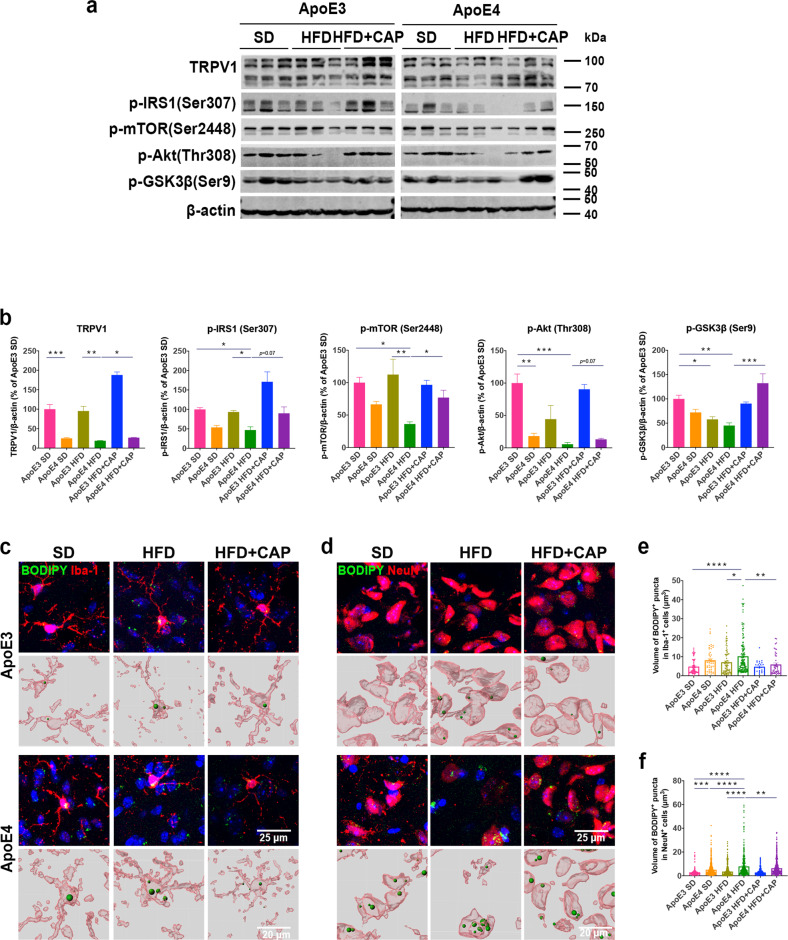
TRPV1 activation reversed lipid droplet accumulation in ApoE4 HFD mice. **a**, **b** Western blot and quantification of TRPV1, p-IRS1 (Ser307), p-mTOR (Ser2448), p-Akt (Thr308), and p-GSK3β (Ser9) levels. **c**, **d** Immunofluorescent images in the upper panel show cortical sections of ApoE3 and ApoE4 mice costained for BODIPY^+^Iba-1^+^ (**c**) or BODIPY^+^NeuN^+^ (**d**) cells. The lower panel shows 3D reconstructions of BODIPY^+^Iba-1^+^ or BODIPY^+^NeuN^+^ cells. **e**, **f** Quantification of BODIPY^+^Iba-1^+^ or BODIPY^+^NeuN^+^ cell percentages in the cerebral cortex. *n* = 3 mice per group (**a**, **c**, **d**). Statistical tests: one-way ANOVA followed by Tukey’s post hoc test (**b**, **e**, **f**). Data represent the mean ± s.e.m. (**b**, **e**, **f**). *p < 0.05, **p < 0.01, ***p < 0.001, ****p < 0.0001. ns, not significant. Scale bars, 25 μm (upper panel), 20 μm (lower panel).

Immunoblotting showed that capsaicin treatment rescued the protein levels of TRPV1 and Akt/mTOR signaling pathway components, including p-IRS1 (Ser307), p-mTOR (Ser2448), and p-Akt (Thr308), in ApoE4 HFD + capsaicin mice compared to those in ApoE4 HFD mice (Fig. 5a, b). Moreover, phosphorylated glycogen synthase kinase (p-GSK3β) (Ser9), downstream of the Akt/mTOR pathway, was significantly upregulated in ApoE4 HFD + capsaicin mice compared with that in ApoE4 HFD mice (Fig. 5a, b). Costaining of BODIPY and Iba-1 or NeuN (Fig. 5c, d) showed a significant decrease in lipid droplet accumulation in both the microglia and neurons of ApoE4 HFD + capsaicin mice compared with that in ApoE4 HFD mice (Fig. 5e, f).

Capsaicin treatment significantly upregulated the expression levels of the autophagy markers Atg7 and LC3B in ApoE4 HFD + capsaicin mice compared to those in ApoE4 HFD mice (Supplementary Fig. 4a, b). Double staining of NeuN and Parkin or LC3B revealed that the percentage of Parkin^+^ or LC3^+^ neurons was significantly increased in the cerebral cortex of ApoE4 HFD + capsaicin mice compared to that in ApoE4 HFD mice (Supplementary Fig. 4c, d). Moreover, colocalization of BODIPY^+^LC3^+^ puncta was increased in ApoE4 HFD + capsaicin mice compared to that in ApoE4 HFD mice (Supplementary Fig. 4e, f).

### TRPV1 activation attenuated the microgliosis phenotype in ApoE4 HFD mice

**Fig. 6:**
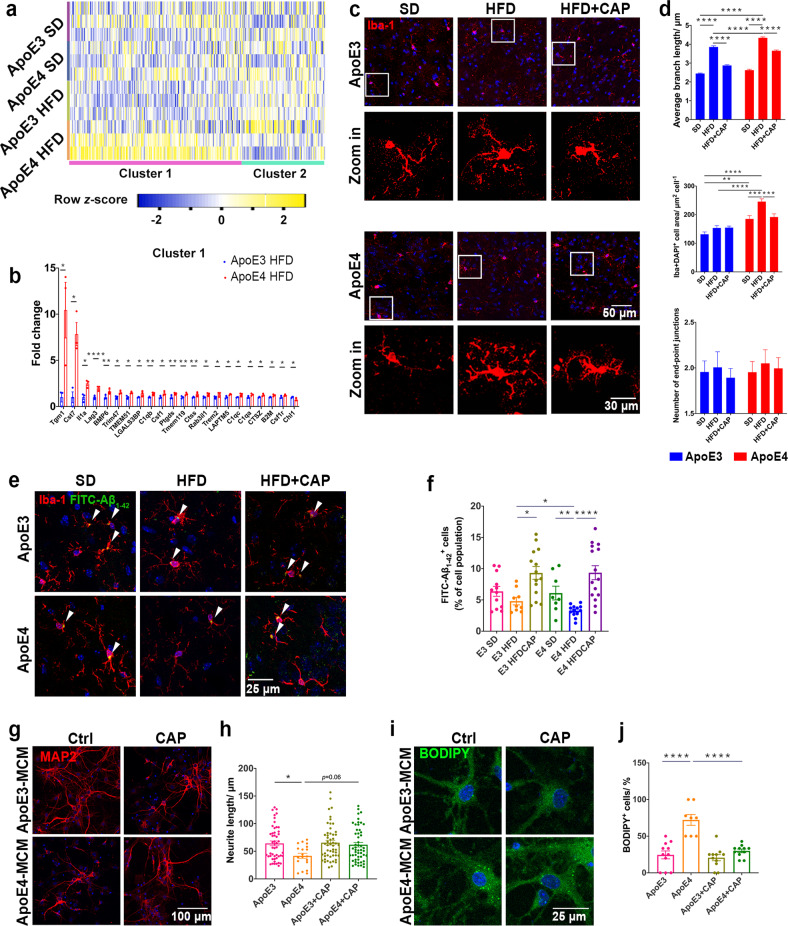
TRPV1 activation attenuated microgliosis and microglial lipid accumulation in ApoE4 HFD mice. **a** Heatmap showing gene expression profiles correlated with microglial proinflammatory reactions (Cluster 1) and normal functions (Cluster 2). **b** Differentially expressed Cluster 1 genes between HFD-fed ApoE3 and ApoE4 mice from the heatmap (**a**). **c** Immunofluorescent images of Iba-1^+^ cells of ApoE3 and ApoE4 mice in the cerebral cortex (upper panel) and amplified single Iba-1^+^ cells (lower panel). **d** Morphological analyses of Iba-1^+^ cells from the immunofluorescence micrographs (**c**). Blue bars indicate ApoE3 mice, and red bars indicate ApoE4 mice. **e** Representative immunofluorescent images of FITC-labeled Aβ_1-42_ and Iba-1^+^ cells of ApoE3 and ApoE4 mice. **f** Quantification of Aβ_1–42_ uptake in Iba-1^+^ cells. **g**, **h** Representative immunofluorescent images and quantification of MAP2 in primary cortical neurons. **i**, **j** Representative immunofluorescent images and quantification of BODIPY^+^ puncta in primary neurons. *n* = 3 mice per group. Statistical tests: two-sided Student’s *t* test (**b**), two-way ANOVA followed by Sidak’s multiple comparison test (**d**), one-way ANOVA followed by Tukey’s post hoc test (**f**, **h**, **j**). Data represent the mean ± s.e.m. *p < 0.05, **p < 0.01, ***p < 0.001, ****p < 0.0001. Scale bars, 50 μm (**c** upper panel), 20 μm (**c** lower panel), 25 μm (**e**).

As brain-resident immune cells, microglia represent a proinflammatory and phagocytosis impairment state with lipid droplet accumulation in the aging mouse brain^29^. Transcriptome profiles of genes related to microglial functions were analyzed. Genes highly expressed in adult homeostasis microglia and proinflammation-related genes significantly affected in experimental autoimmune encephalomyelitis, APP/PS1, and SOD1-mutant mouse brains were selected for analysis^43–45^. Our results revealed markedly upregulated expression of proinflammatory genes (Cluster 1) and downregulated expression of normal cell function genes (Cluster 2) in ApoE4 HFD mouse brains compared that in ApoE3 HFD mice (Fig. 6a, b). Changes in microglial inflammatory responses also reflected morphological alterations, which have been reported in human AD patients and several AD mouse models^46–48^. Morphometric analysis of microglia in the cerebral cortex revealed that the microglial branch length and the Iba-1^+^ cell area of ApoE4 HFD mice were significantly increased compared to those of ApoE3 HFD mice (Fig. 6c, d). The number of microglial end-point junctions also showed a growth trend in ApoE4 HFD mice (Fig. 6c, d). Microglia of ApoE4 HFD + capsaicin mice exhibited a significantly more activated state than those of ApoE4 HFD mice, including shorter microglial branch length, smaller Iba-1^+^ cell area, and fewer microglial end-point junctions.

To determine whether TRPV1 activation rescued impaired microglial phagocytic activity induced by ApoE4, a microglial phagocytosis assay was performed. FITC-Aβ_1–42_ was sterotactically administered into the hippocampus of ApoE mice to assess phagocytic activity in vivo. Phagocytosis assays showed significantly more colocalization of Iba-1^+^ microglia and FITC-Aβ_1–42_ in ApoE4 HFD + capsaicin mice than in ApoE4 HFD mice (Fig. 6e, f).

BV2 cells were pretreated with 10 μM capsaicin and then exposed to 150 nM human recombinant ApoE3 or ApoE4 for 24 h. Afterward, ApoE3-MCM, ApoE4-MCM, ApoE3-MCM + capsaicin, and ApoE4-MCM + capsaicin were collected. Primary cortical neurons were exposed to MCM for 24 h, and neurite growth and lipid droplet accumulation were evaluated. Immunofluorescent images of MAP2^+^ primary neurons showed that the neurite length of the ApoE4-MCM + capsaicin group neurons was longer than that of the ApoE4-MCM group neurons, suggesting that TRPV1 activation rescued impaired neuronal outgrowth induced by ApoE4 microglia (Fig. 6g, h). BODIPY staining showed less lipid droplet accumulation in ApoE4-MCM + capsaicin group neurons than in ApoE4-MCM cells (Fig. 6i, j).

### TRPV1 activation attenuated microglial phagocytosis of synapses in ApoE4 HFD mice

**Fig. 7:**
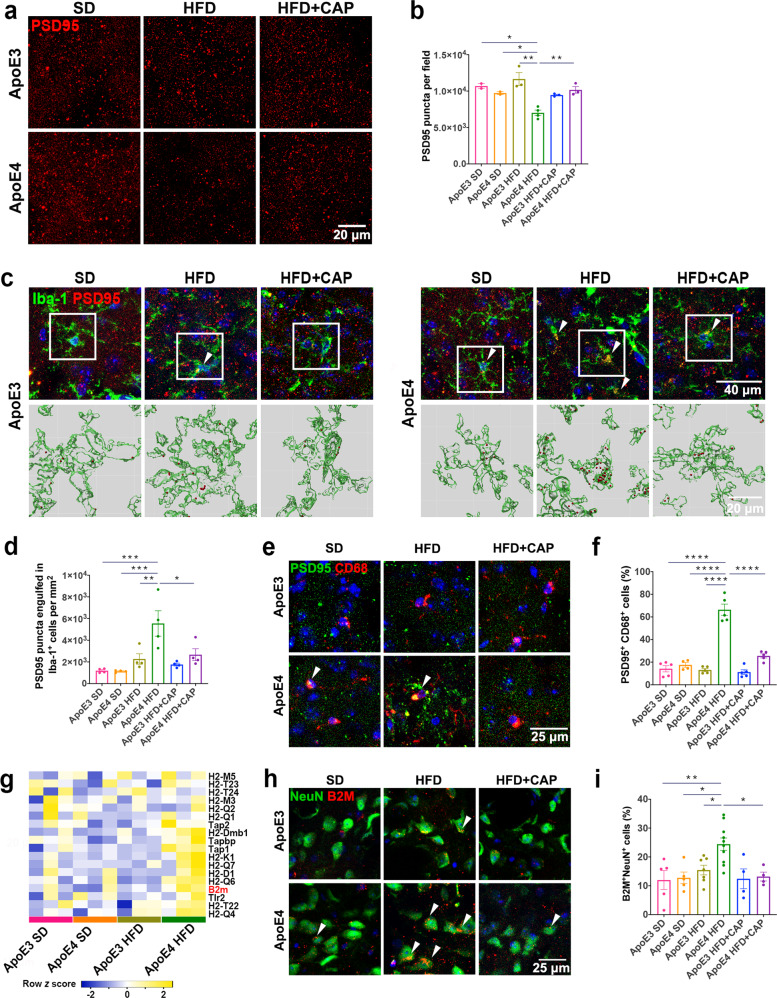
TRPV1 activation attenuated microglial phagocytosis of synapses in ApoE4 HFD mice. **a**, **b** Representative immunofluorescent images and quantification of PSD95 puncta in the cortex of ApoE3 and ApoE4 mice. **c**, **d** Representative immunofluorescence micrographs and quantification of PSD95 engulfed in Iba-1^+^ microglia (upper panel) and 3D reconstruction (lower panel) showing the volume of Iba-1 (green) and engulfed PSD95 puncta (red) in the cortex of ApoE3 and ApoE4 mice. **e**, **f** Representative immunofluorescent images and quantification of PSD95 (green) and CD68 (red) in the cortex of ApoE3 and ApoE4 mice. **g** Heatmap showing gene expression profiles enriched in the MHC-1 pathway of HFD-fed ApoE3 and ApoE4 mice. **h**, **i** Representative immunofluorescent images and quantification of NeuN^+^ and B2M^+^ cells in the cortex of ApoE3 and ApoE4 mice. *n* = 3 mice per group. Statistical tests: one-way ANOVA followed by Tukey’s post hoc test (**b**, **d**, **f**, **i**). Data represent the mean ± s.e.m. *p < 0.05, **p < 0.01, ***p < 0.001, ****p < 0.0001. Scale bars, 20 μm (**a**, **c** upper panel), 10 μm (**c** lower panel), 25 μm (**f**, **h**).

Our previous results showed that neuron loss was significantly increased in ApoE4 HFD mice compared with ApoE3 HFD mice, while TRPV1 activation rescued this loss in ApoE4 HFD + capsaicin mice (Fig. 4h, i). To investigate the effects of TRPV1 on neuronal synapses, the postsynaptic marker PSD95 was used for staining. Confocal images depicted a reduction in PSD95 puncta in ApoE4 HFD mice compared with ApoE3 HFD mice. Capsaicin treatment rescued PSD95 puncta loss in ApoE4 HFD mice (Fig. 7a, b). In terms of the effect of ApoE4 on augmenting microglial engulfment of synapses by upregulating neuronal major histocompatibility complex 1 (MHC-I) expression^1^, we hypothesized that TRPV1 activation rescued synapse loss by downregulating microglial engulfment. Double staining with PSD95 and Iba-1 showed that TRPV1 activation significantly downregulated microglial engulfment of synapses in ApoE4 HFD + capsaicin mice compared to that in ApoE4 HFD mice (Fig. 7c, d).

Brain sections were costained with PSD95 and CD68 to investigate whether PSD95 puncta were phagocytosed by microglia (Fig. 7e, f). Increased colocalization of PSD95 puncta with CD68-positive vesicles was observed in ApoE4 HFD mice compared with ApoE3 HFD mice, while less colocalization was observed in ApoE4 HFD + capsaicin mice compared with ApoE4 HFD mice.

We further hypothesized that PSD95 puncta loss was induced by increasing neuronal MHC-I expression in ApoE4 HFD mice, as previously reported^1^. RNA-seq depicted upregulation of MHC-I gene expression (*H2-M5*, *H2-T23*, *H2-T24*, *H2-M3*, *H2-Q2*, *H2- Q1*, *Tap2*, *H2-Dmb1*, *Tapbp*, *Tap1*, *H2-K1*, *H2-Q7*, *H2-D1*, *H2-Q6*, *B2m*, *Tlr2*, *H2-T22*, *and H2-Q4*) in ApoE4 HFD mice compared with ApoE3 HFD mice (Fig. 7g). Double staining with NeuN and beta-2 microglobulin (B2M), a regulator of functional MHC-I expression, revealed increased neuronal B2M expression, indicating upregulation of the MHC-I pathway in ApoE4 HFD mice. TRPV1 activation significantly decreased neuronal B2M expression in ApoE4 HFD + capsaicin mice (Fig. 7h, i).

### TRPV1 gene deletion exacerbated recognition impairment and tau pathology in ApoE4 mice

**Fig. 8:**
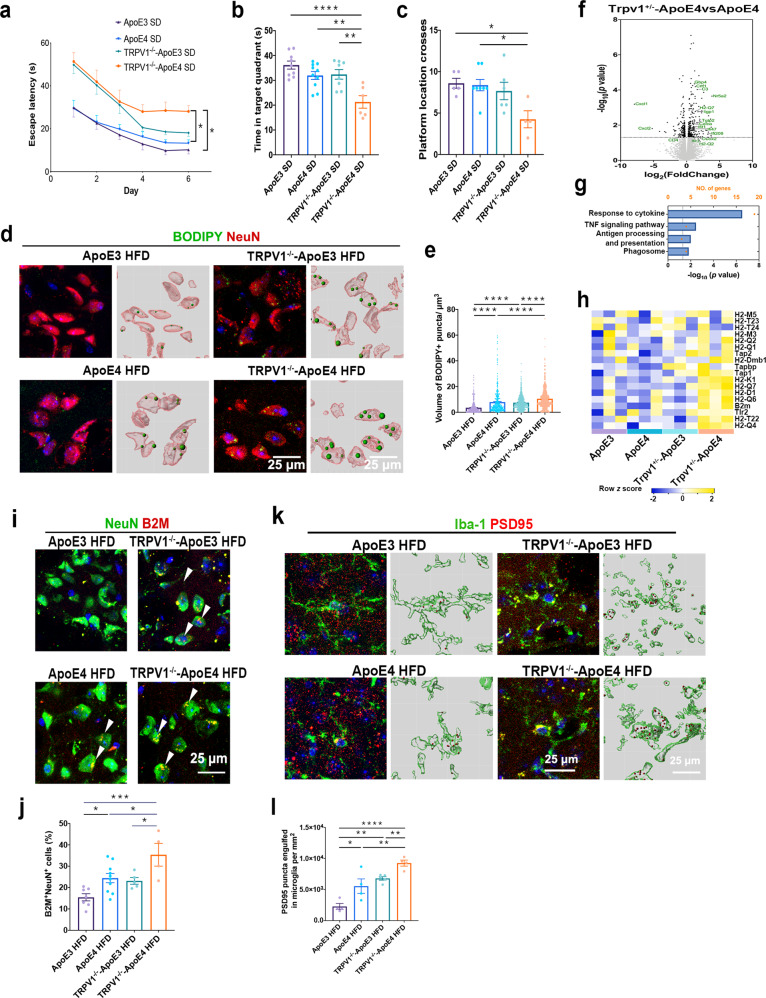
TRPV1 genetic deficiency exacerbated recognition impairment and neuronal lipid droplet accumulation in ApoE4 mice. **a**–**c** MWM for TRPV1^−/−^-ApoE3 and TRPV1^−/−^-ApoE4 mice. Line chart shows MWM escape latency (time to find the hidden platform) for six consecutive days (**a**). Histograms show time spent in the target quadrant and times crossing the platform location in the MWM probe trial (**b**, **c**). **d** Immunofluorescent images in the left panel show cortical sections of ApoE3 and ApoE4 mice stained for BODIPY and NeuN. The right panel shows 3D reconstructions of BODIPY^+^NeuN^+^ neuronal soma. **e** Volume of BODIPY^+^ puncta in NeuN^+^ cells in the cerebral cortex. **f** Volcano plot showing differentially expressed genes between ApoE4 and TRPV1^−/−^-ApoE4 mouse brains. Dotted lines indicate the *p* < 0.05 cutoff (*n* = 3 mice per group; two-sided Student’s *t* test, Benjamini‒Hochberg FDR). **g** Enrichment pathway analysis of genes differentially expressed in ApoE4 and TRPV1^−/−^-ApoE4 mouse brains. **h** Heatmap showing gene expression profiles enriched in the MHC-I pathway in TRPV1^−/−^-ApoE3 and TRPV1^−/−^-ApoE4 mice. **i**, **j** Representative immunofluorescent images and quantification of NeuN^+^ and B2M^+^ cells in the cortex of ApoE3 and ApoE4 mice. **k** Representative immunofluorescence micrographs of ApoE3 and ApoE4 mouse cerebral cortex stained for Iba-1 and PSD95 (left panel) and 3D reconstruction (right panel) showing the volume of Iba-1 (green) and engulfed PSD95 puncta (red). **l** Quantification of PSD95 puncta numbers and Iba-1 engulfed from immunofluorescent images. *n* = 3 mice per group. Statistical tests: one-way ANOVA followed by Tukey’s post hoc test (**b**, **c**, **e**, **g**, **j**, **l**) and two-way ANOVA followed by Tukey’s post hoc test (**a**). Data represent the mean ± s.e.m. *p < 0.05, **p < 0.01, ****p < 0.0001. Scale bars, 25 μm.

To further investigate the effect of TRPV1 on neuronal lipid metabolism dysfunction, TRPV1-deficient ApoE3 and ApoE4 (TRPV1^−/−^-ApoE3 and TRPV1^−/−^-ApoE4) mice were generated, and the MWM was performed at 6 months of age. TRPV1^−/−^-ApoE4 mice showed a significantly longer escape latency than ApoE4 mice during the MWM training session (Fig. 8a). In the probe trial of the MWM, TRPV1^−/−^-ApoE4 mice spent a significantly shorter period of time in the target quadrant and had fewer platform location crosses than ApoE4 mice (Fig. 8b, c).

Three-month-old TRPV1^−/−^-ApoE4 mice were also fed a HFD for 3 months. Costaining with BODIPY and NeuN showed significantly larger lipid droplets in the neuronal somata of TRPV1^−/−^-ApoE4 HFD mice than in those of ApoE4 HFD mice, indicating that genetic deletion of TRPV1 resulted in increased lipid droplet accumulation in the neurons of ApoE4 mice (Fig. 8d, e).

RNA-seq was performed on TRPV1 heterozygous ApoE4 (TRPV1^+/−^-ApoE4) mouse brains to investigate changes induced by TRPV1 deficiency in ApoE4 mice. Unsupervised cluster analysis segregated ApoE4 from TRPV1^+/−^-ApoE4 mice and revealed prominent differences between their transcriptomes; 480 differentially expressed genes were detected (Fig. 8f). Enrichment analysis showed that inflammation and phagosome pathways, including response to cytokines, TNF signaling, antigen processing and presentation, and phagosomes, were enriched in TRPV1^+/−^-ApoE4 mice (Fig. 8g). Consistent with the results of Fig. 8h, RNA-seq revealed upregulation of MHC-I gene expression in TRPV1^+/−^-ApoE4 mice compared with ApoE4 mice (Fig. 8h). Double staining with NeuN and B2M revealed that TRPV1 depletion significantly increased neuronal B2M expression in TRPV1^−/−^-ApoE4 HFD mice compared with ApoE4 HFD mice (Fig. 8i, j).

Costaining of Iba-1 and PSD95 was also performed to investigate whether genetic deletion of TRPV1 increased microglial engulfment of synapses in TRPV1^−/−^-ApoE4 HFD mice (Fig. 8k, l). Our results showed that significantly more Iba-1^+^ cells engulfed PSD95 puncta in TRPV1^−/−^-ApoE4 HFD mice than in ApoE4 HFD mice (Fig. 8k, l). Overall, genetic TRPV1 deletion led to the engulfment of more synapses in microglia via upregulation of neuronal MHC-I expression in ApoE4 mice.

**Fig. 9:**
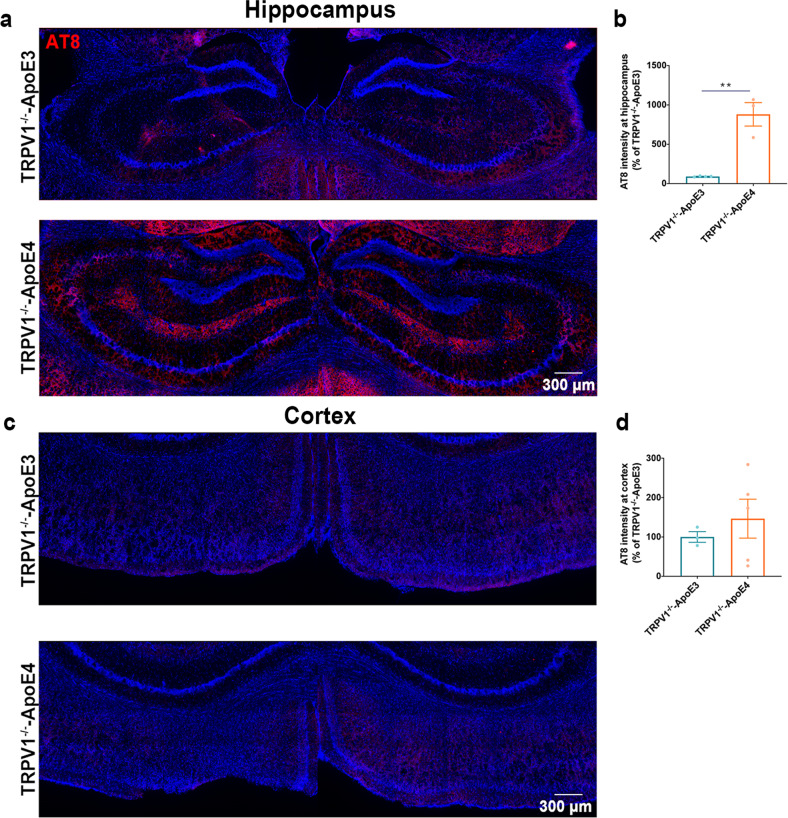
TRPV1 genetic deficiency exacerbated ApoE4-induced tau pathology. **a**–**d** Phosphorylated tau-covered area in the cortex and hippocampus of TRPV1^−/−^-ApoE3 and TRPV1^−/−^-ApoE4 mice. (*n* = 3, biological replicates). Statistical tests: two-sided Student’s *t* test (**b**, **d**). Data represent the mean ± s.e.m. **p < 0.01. Scale bars, 300 μm.

The expression of genes associated with autophagy was markedly downregulated in TRPV1^−/−^-ApoE4 mice compared with TRPV1^−/−^-ApoE3 mice (Supplementary Fig. 5a, b). The expression of genes associated with cholesterol biosynthesis was markedly upregulated in TRPV1^−/−^-ApoE4 mice (Supplementary Fig. 5c, d). Immunofluorescent images of phosphorylated tau (AT-8) revealed significant upregulation in the hippocampus of TRPV1^−/−^-ApoE4 mice compared with TRPV1^−/−^-ApoE3 mice (Fig. 9a–d).

## Discussion

Here, we report that the *APOE4* allele leads to neuronal bioenergetic function impairment, including that of lipid and energy metabolism, in human ApoE-targeted replacement mice. These changes resulted in neuronal lipid droplet accumulation-induced impairment of neuronal autophagy. We showed that TRPV1 activation with capsaicin reversed neuronal lipid droplet accumulation and autophagy impairment in primary neurons in vitro. Capsaicin also rescued memory impairment, tau pathology, and neuronal autophagy dysfunction in ApoE4 HFD mice. Activation of TRPV1 decreased neuronal lipid droplet accumulation and induced upregulation of microglial phagocytosis of synapses in ApoE4 HFD mice. TRPV1 gene deficiency exacerbated recognition and neuronal immune homeostasis impairment in ApoE4 HFD mice.

Here, we report that ApoE4 disrupted the neuronal lipidome. Lipidomics and unbiased genome-wide screens were combined with functional and genetic characterization in mice to demonstrate that the presence of the *APOE4* allele led to lipid homeostasis impairment and immune dysfunction. AD patients carrying the *APOE4* allele accumulated more lipid droplets in neurons and microglia, but not astrocytes, than normal individuals. Lipid metabolic LC‒MS analysis of the peripheral plasma, which has been shown to reflect lipid metabolic changes in AD, revealed downregulated medium- and long-chain FAs in ApoE4 mice compared with ApoE3 mice (Fig. 1c–e). Enrichment analysis showed that pathways related to the inflammatory response, energy metabolic process, cholesterol metabolism, and insulin resistance were enriched in the ApoE4 mouse brain (Fig. 1i).

ApoE4 impaired bioenergetic function and neurite outgrowth in primary neurons. We evaluated the effects of the ApoE isoform on oxidative phosphorylation in primary neurons. ApoE4 neurons exhibited lower basal respiration and maximal respiration than ApoE3 controls (Fig. 3j). As a key metric of neuronal function, the outgrowth of neurites is energy dependent. As shown in Fig. 3k, neurite length was significantly lower in E4 versus E3 neurons (Fig. 3k). Importantly, phosphorylation of the energetic metabolism pathway proteins IRS1, mTOR, Akt, and AMPK was reduced by more than 50% in ApoE4 neurons compared with the ApoE3 control (Fig. 3a). Collectively, these data suggested that the *APOE4* allele in neurons dampened mitochondrial oxidative phosphorylation, neurite growth, and synaptic density.

The HFD aggravated impaired energy metabolism, leading to neuronal dysfunction in ApoE4 mice. Marschallinger et al. reported that lipid droplet accumulation in microglia represented a dysfunctional state in the aging brain, which included impaired phagocytosis, a proinflammatory status, and high levels of ROS^49^. Phagocytosis was tested as shown in Fig. 7, and reduced colocalization of Iba-1^+^ microglia and FITC-Aβ_1–42_ was observed in ApoE4 HFD mice compared to that in ApoE3 HFD mice (Fig. 7e).

Oxidized low-density lipoprotein-induced lipid accumulation was ameliorated by TRPV1 agonizts in macrophages. TRPV1 activation promoted cholesterol efflux via upregulation of the ATP-binding cassette (ABC) transporters ABCA1 and ABCG1. The upregulation of ABCA1 and ABCG1 occurred mainly through liver X receptor α-dependent transcription in macrophages^50^. Capsaicin rescued oxLDL-induced autophagy impairment and autophagy‒lysosome pathway induction in VSMCs. Capsaicin inhibited VSMC foam cell formation through autophagy induction via the AMPK signaling pathway in oxLDL-treated VSMCs^14^. Our study showed that TRPV1 activation reversed neuronal autophagy impairment (Fig. 3f, h) and lipid droplet accumulation (Figs. 3c and 5c).

Neuronal ApoE was reported to elicit AD-related tau pathologies through neuronal MHC-I^1^. This study provides evidence that neuronal ApoE-induced overexpression of MHC-I might serve as an injured neuronal ‘eat-me’ signal to brain immune microglia and CD8^+^ T cells. TRPV1 regulated microglial proinflammation and dysfunction of phagocytosis (Fig. 7a, e), and TRPV1 activation rescued memory impairment and neuronal dysfunction in ApoE4 mice. TRPV1 gene deficiency exacerbated recognition and neuronal homeostasis impairment in ApoE4 HFD-fed mice. These data indicate that genetic TRPV1 deletion leads to increased microglial engulfment of synapses, which was induced by upregulation of neuronal MHC-I expression in ApoE4 mice (Fig. 8h).

The limitation of our study was that five-month-old ApoE3 and ApoE4 HFD mice were used to monitor whether TRPV1 activation could rescue neuronal lipid metabolism in vivo. It was reported that neuronal loss and cognitive deficits were found in 12-month-old APOE4 mice, which are relatively old mice and consistent with human LOAD epidemiology^1,51–53^. Further study should focus on the effects of capsaicin in regulating ApoE4-disrupted intracellular lipid homeostasis in the aging ApoE4 mouse brain.

Overall, our study suggests that TRPV1 regulation of neuronal lipid metabolism and autophagy homeostasis could be a therapeutic approach to alleviate the consequences of the ApoE4 allele.

## Availability of data and material

The authors declare that the data supporting the findings of this study are available from the corresponding author upon reasonable request.

## Supplementary information

Supplement figure and legend
