# Supplementary material for: TRPV1 regulates ApoE4-disrupted intracellular lipid homeostasis and decreases synaptic phagocytosis by microglia
Source: Exp Mol Med. 2023 Feb 1;55(2):347–63. doi: 10.1038/s12276-023-00935-z (PMC9981624; doi:10.1038/s12276-023-00935-z)
Supplement: Supplementary file 1 — Supplement figure and legend [file 12276_2023_935_MOESM1_ESM.pdf]

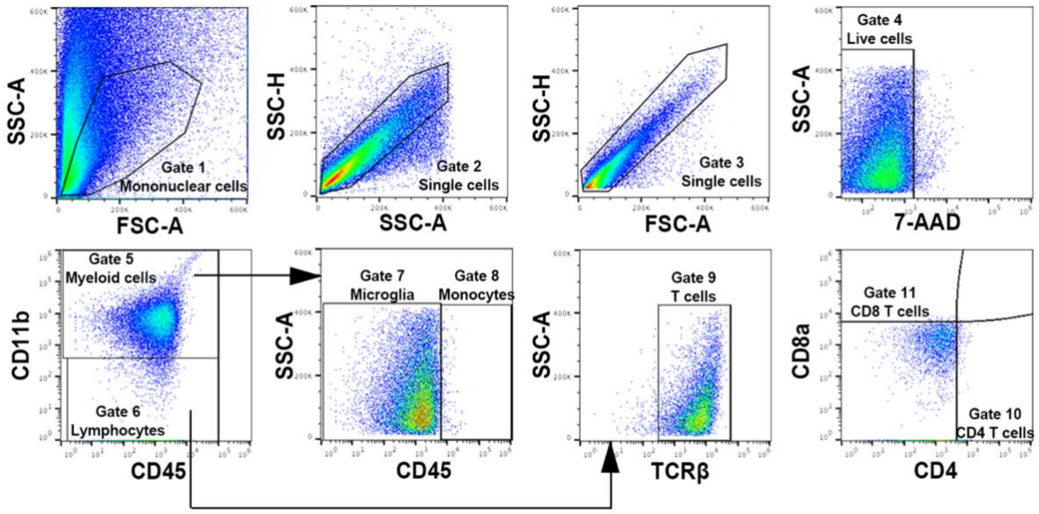

Supplementary Fig. 1 Gating strategy for flow cytometric analysis of ApoE3 and ApoE4 mice brain.

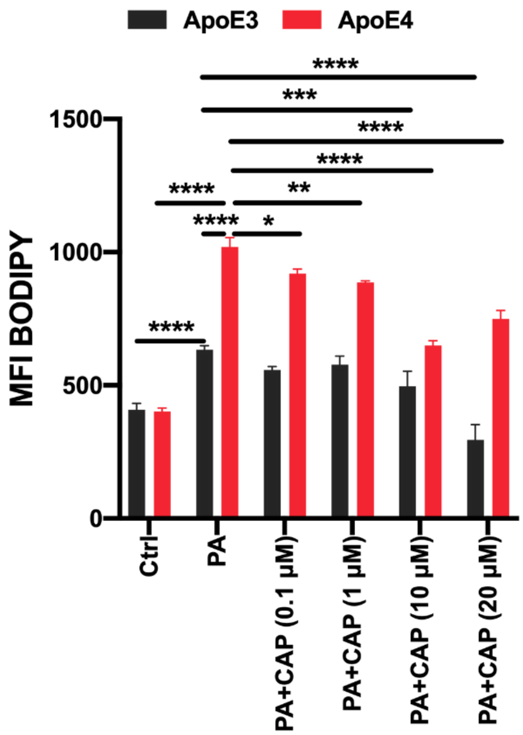

Supplementary Fig. 2 Capsaicin decreased the BODIPY^{+} cells in ApoE4 BV2 cells in a dose dependent manner.

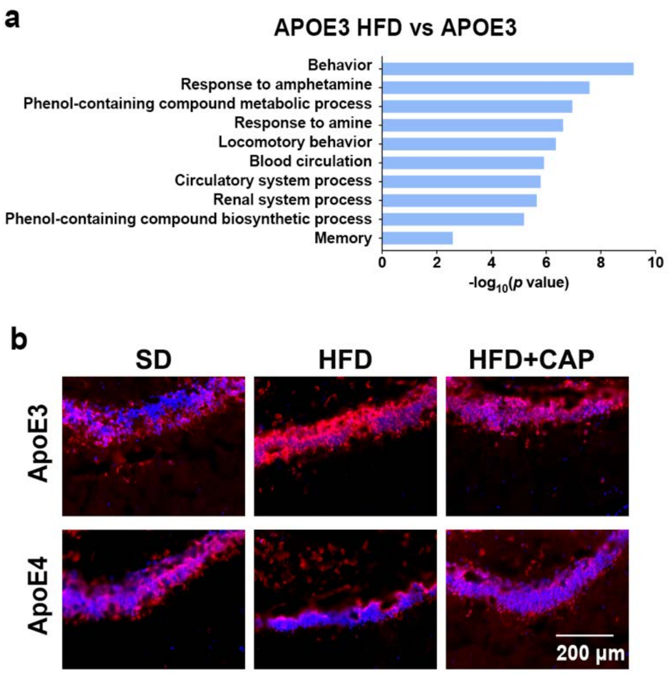

Supplementary Fig. 3 Top 10 GO enrichment pathways of genes differentially expressed between ApoE3 HFD and ApoE3 SD mice.

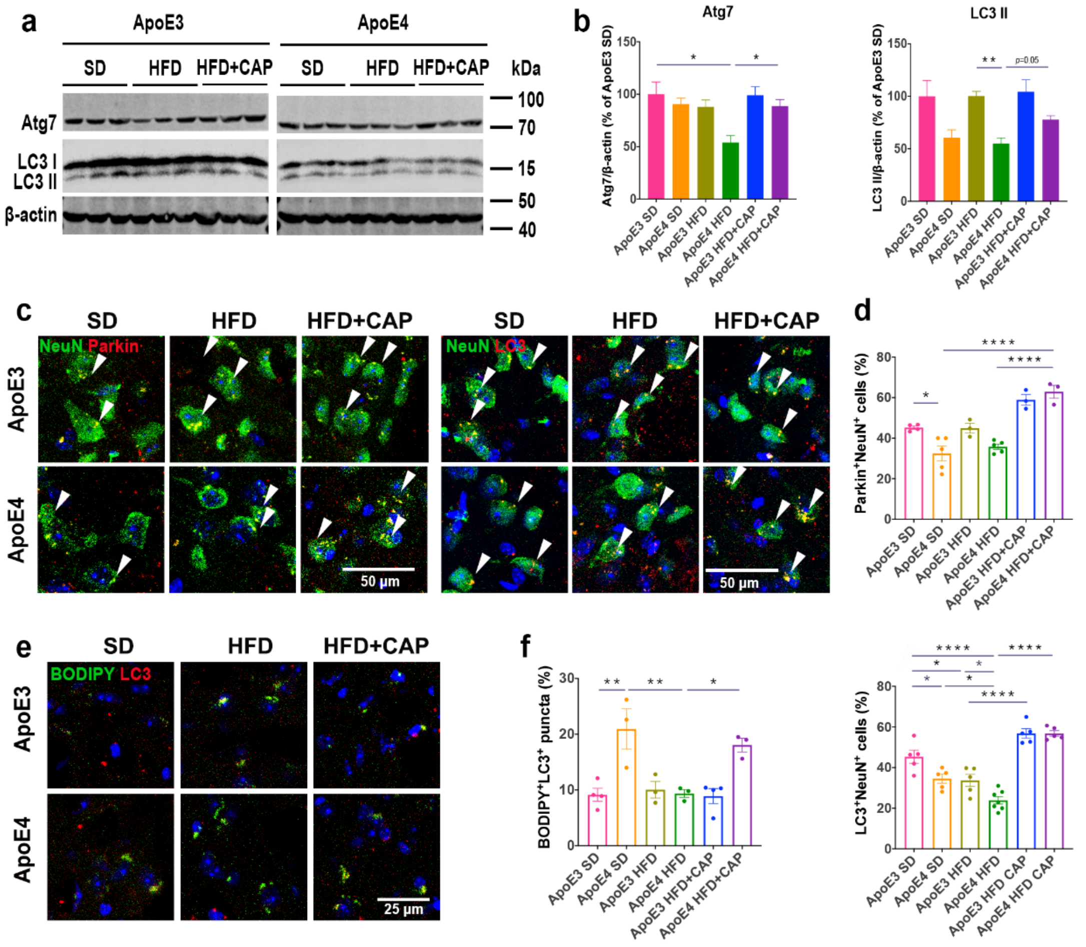

Supplementary Fig. 4 TRPV1 activation reversed neuronal autophagy impairment in ApoE4 HFD-fed mice.

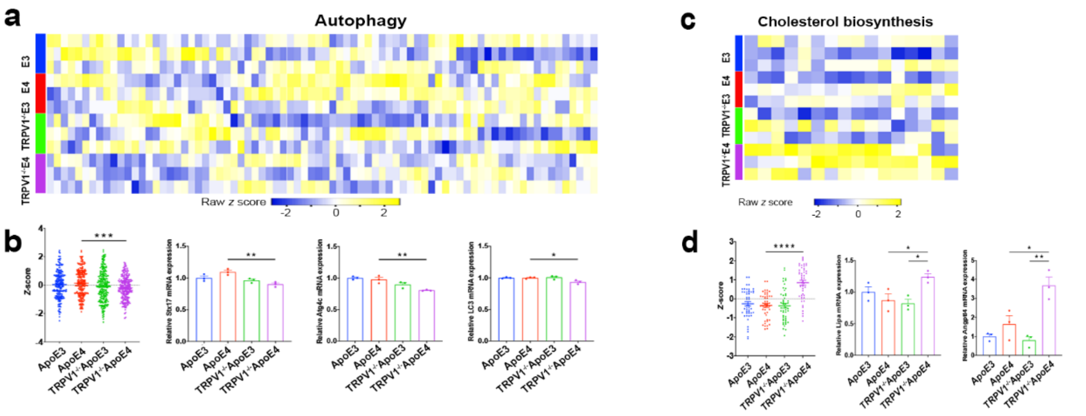

Supplementary Fig. 5 TRPV1 genetic deficiency exacerbated ApoE4-induced tau pathology.

Supplementary Figure legends

Supplementary Fig. 1 Gating strategy for flow cytometric analysis of ApoE3 and ApoE4 mice brain.

Mononuclear cells were isolated from ApoE3 and ApoE4 mice brain, and were surface stained with CD45, CD11b, MHC-II, TCRβ, CD4, and CD8a antibodies. 7-AAD cell viability dye was applied for separating live cells from all single cells. Cells were gated at the first for mononuclear cell population (Gate 1), single cell population (Gate 2 and 3), and following gated for live cell population (Gate 4). Then $CD11b^{high}CD45^{+}$ cells (Gate 5) and $CD11b^{low}CD45^{+}$ cells (Gate 6) were separated. Myeloid cell population were subsequently separated into $CD11b^{high}CD45^{low}$ microglia (Gate 7 population) and $CD11b^{high}CD45^{high}$ monocytes (Gate 8 population), while lymphocyte population were gated for $TCR\beta^{high}$ cell (Gate 9). Finally, $TCR\beta^{high}$ cell were separated into $CD4^{high}CD8a^{low}$ CD4 T cells (Gate 10 population) and $CD4^{low}CD8a^{high}$ CD8 T cells (Gate 11 population).

Supplementary Fig. 2 Capsaicin decreased the $BODIPY^{+}$ cells in ApoE4 BV2 cells in a dose dependent manner.

Flow cytometry showed 0.1, 1, 10, 20 μM capsaicin decreased the $BODIPY^{+}$ cells in ApoE4 BV2 cells in a dose dependent manner. Statistical tests: one-way ANOVA followed by Tukey's post hoc test. Data represent the mean ± s.e.m. $^{*}p < 0.05$, $^{**}p < 0.01$, $^{***}p < 0.001$, $^{****}p < 0.0001$.

Supplementary Fig. 3 Top 10 GO enrichment pathways of genes differentially expressed between ApoE3 HFD and ApoE3 SD mice.

(a) Top 10 GO enrichment pathways of genes differentially expressed between ApoE3 HFD and ApoE3 SD mice. (b) Thickness of the granule cell layer of the dentate gyrus. Scale bars, 200 μm.

Supplementary Fig. 4 TRPV1 activation reversed neuronal autophagy impairment in ApoE4 HFD-fed mice.

(a, b) Western blot and quantification of Atg7 and LC3 in cerebral cortex tissues. (c, d) Representative immunofluorescent images and quantification of $Parkin^{+}NeuN^{+}$ and $LC3^{+}NeuN^{+}$ cell percentages in the cerebral cortex of ApoE3 and ApoE4 mice. (e, f) Representative immunofluorescent images and quantification of BODIPY and LC3 in the cerebral cortex. n = 3 mice per group. Statistical tests: one-way ANOVA followed by Tukey's post hoc test (b, d, f). Data represent the mean ± s.e.m (b, d, f). $^{*}p < 0.05$, $^{**}p < 0.01$, $^{***}p < 0.001$, $^{****}p < 0.0001$. Scale bars, 50 μm (c), 25 μm (e).

Supplementary Fig. 5 TRPV1 genetic deficiency exacerbated ApoE4-induced dysfunction of autophagy and cholesterol biosynthesis.

(a-d) Heat map representing relative expression levels of genes associated with autophagy (a, b) and cholesterol biosynthesis (c, d). Statistical tests: one-way ANOVA followed by Tukey's post hoc test (b, d). Data represent the mean ± s.e.m. $^{*}p < 0.05$, $^{**}p < 0.01$, $^{***}p < 0.001$, $^{****}p < 0.0001$.
